# Quantitative analysis of polystyrene microplastic and styrene monomer released from plastic food containers

**DOI:** 10.1016/j.heliyon.2023.e15787

**Published:** 2023-04-25

**Authors:** Jiae Wang, Jieun Lee, Eilhann E. Kwon, Sanghyun Jeong

**Affiliations:** a Department of Environmental Engineering, Pusan National University, Busan, 46241, South Korea; b Institute for Environmental and Energy, Pusan National University, Busan, 46241, South Korea; c Department of Earth Resources and Environmental Engineering, Hanyang University, Seoul, 04763, South Korea

**Keywords:** Plastic container, Microplastics, Polystyrene, Styrene, Fourier-transformed infrared spectroscopy, Simultaneously exposed pollutant

## Abstract

Since the COVID-19 outbreak, the use of disposable plastics has rapidly increased along with the amount of plastic waste. During fragmentation, microplastics and other chemical substances contained in plastics are released. These then enter humans through food which could be problematic considering their hazardous potential. Polystyrene (PS), which is widely used in disposable containers, releases large amounts of microplastics (MPs), but no studies have investigated the release mechanisms of PS-MPs and simultaneously exposed contaminants. Therefore, in this study, the effects of pH (3, 5, 7, and 9), temperature (20, 50, 80, and 100 °C), and exposure time (2, 4, 6, and 8 h) on MPs release were systematically examined. A quantitative/qualitative study of MPs and styrene monomers was performed using microscopy-equipped Fourier-transformed infrared spectroscopy and gas chromatography-mass spectrometry. The release of PS-MPs (36 items/container) and simultaneously exposed pollutants (SEP), such as ethylene glycol monooleate (EGM), was highest at pH 9, 100 °C, and 6 h, which was proportional to the test temperature and time. Under the same conditions, 2.58 μg/L of styrene monomer migrated to the liquid food simulants. The fragmentation was proceeded by oxidation/hydrolysis and accelerated by increased temperature and exposure time. The strong positive correlation between PS-MPs and SEPs releases at pH and temperature indicates that PS-MPs and SEPs follow the same release process. However, a strongly negative correlation between PS-MPs and styrene monomers at the exposed time shows that styrene migration does not follow the same release process, but does its partition coefficient.

## 1 Introduction

Plastics are used in various products, and this use has intensified as plastics have versatile physicochemical properties compared to the conventional materials such as glass, metal, and wood. The global consumption of plastics has increased by more than 200 times from 1.7 billion kg in the 1950s to over 367 billion kg in the 2020s [1]. In particular, the use of disposable (or single-use) plastics has increased tremendously since the COVID-19 pandemic [2]. In 2019, 49% of global plastics were produced as disposable items [3]. COVID-19 changed the lifestyle and dietary habits of consumers [4] and increased the use of single-use plastics in food packaging, fast food delivery, and take-out food. Moreover, some policies that banned the use of plastic bags or single-use plastics were suspended during the pandemic [[5], [6], [7]]. Hence, the role of plastics in food packaging has been emphasized by the COVID-19 pandemic [8]. In 2020, plastic packaging accounted for the largest portion (40.5%) of the total plastic demand in Europe [1]. Therefore, concerns regarding the safety of plastics have increased substantially [9].

In daily life, commercial plastic packages or containers that are made of polystyrene (PS), polypropylene (PP), or polyethylene (PE) are generally used [10]. PS, which is the second-most produced plastic after PE [11], is widely used in disposable (or single-use) food containers, such as cup noodles, disposable lunch boxes, and yoghurt boxes. Plastics are broken down mechanically, chemically, or thermally and can form microplastics (MPs) via several mechanisms [12]. According to the United States National Oceanic and Atmospheric Administration (NOAA), MPs are defined as plastic particles less than 5 mm in size [13,14].

The biggest concern regarding MPs released from plastics include human exposure and the subsequent adverse effects on their health [15]. Human exposure to MPs can occur through several routes [16]; however, most attention has been paid to food containers because they are in direct contact with humans [17,18]. When humans are exposed to MPs via ingestion or inhalation, these MPs can elicit a local immune response [19]. MPs have been detected in single-use plastic containers, water bottles, and infant-feeding bottles [12]. Furthermore, MPs have been found in human stool, hair, facial skin, hand skin, saliva, lung tissue, and even placenta [15,[20], [21], [22]]. Recent studies have estimated that the annual exposure via inhalation and ingestion is 74,000–121,000 MPs per person [23]. Hence, there is a growing concern regarding the risks associated with the ingestion of MPs released from PS containers (PS-MPs) [17,24]. Zhang et al. [25] found that the exposure of pregnant female mice to PS-MPs of various sizes resulted in metabolic disturbances in the offspring. Their analysis suggested that MPs exposure in fish livers induces alterations in the metabolic profile, interferes with lipid and energy metabolism, and reveals PS-MP toxicity [26].

Plastic materials and their associated chemicals may pose risks to human health [27]. Some previous studies detected MPs and volatiles released from PS food containers. For example, J’Bari et al. [28] investigated the fate of MPs and styrene (monomer of PS) from PS fragments and detected the migration of MPs and styrene into tested food simulants from typical containers using the Nile Red staining method and high-performance liquid chromatography. PS was obtained by polymerizing styrene monomers [29]. PS is known to release styrene, which has several negative health effects [30].

Styrene is a toxic substance that irritates the mucous membranes of the nose and throat, and it causes wheezing and coughing if inhaled repeatedly for a long period [31]. Styrene also causes neurological disorders with toxic effects on the liver and acts as a depressant of the central nervous system [32,33]. The World Health Organization (WHO) has classified styrene as a possible human carcinogen. Other studies have shown that styrene exhibits oestrogen-like activity by binding to estrogen receptors [34,35]. The transfer of styrene from PS to foodstuffs has been extensively studied [36,37]. Migration of styrene monomer from PS containers/packaging in contact with food was investigated and quantified at different temperature and food simulants [[38], [39], [40]].

However, most previous studies on release tests in PS-made food containers have focused on styrene migration and not on PS-MPs release, which can be easily released from broken plastics. In this case, simultaneously exposed pollutants (SEPs) such as plasticizer can also emerge from plastic materials. This should be investigated to understand the fate of MPs and SEPs in plastics under different pH and temperature condition. Because exposure to hazardous substances leached from plastics is ubiquitous and the toxic effects of such exposures are of concern, systematic research on the identification of these substances is required. However, only a few studies have identified PS-MPs and styrene that can leach from PS food containers.

In this study, MPs and styrene leaching from PS food containers at different pH values, temperatures, and times was analyzed. The release of MPs was investigated at different pH values to simulate the fact that PS containers are commonly used in daily life under different pH conditions. A microscope equipped with Fourier-transformed infrared (μ-FTIR) spectroscopy was used to detect MPs, and gas chromatography-mass spectrometry (GC-MS) was used to identify and quantify styrene. Primarily, this study aimed to examine the correlation between MPs and styrene monomer released from PS food containers. The release conditions of MPs and styrene (or its monomer) and the correlations between them are fundamental data that can be used for the safety and human risk assessment of plastic containers.

## 2 Material and methods

### 2.1 Materials

To assess the release of MPs from a plastic container, a commercial PS food container (instant noodle container) was purchased six months prior to the expiration date. An entire part of the PS container—without cutting to it a uniform size—was used for this test. This part was moderately washed several times with deionized (DI) water. The PS food container was white in color and did not contain printed images (Fig. S1(a)). The chemical composition of the PS food containers was identified using attenuated total reflection (ATR)-FTIR spectroscopy (Nicolet iN50, Thermo Fisher Scientific, US). The spectral range of the sample was set to 4000–500 cm^−1^ with 16 scans, and the spectral resolution was 4 cm^−1^ as measured using the mercury cadmium telluride (MCT) detector. The spectrum obtained for the PS container was compared with that of the PS reference in the database of the Thermo Scientific Infrared spectral library. All samples showed more than 80% matching with PS (Fig. S(1b)).

To test the release at different pH values, solutions with pH 3, 5, 7, and 9 were prepared by adding a few drops of (or dropping) 0.1 N–HCl (Junsei, Japan) and 0.1N–NaOH (Daejung, Korea). pH was measured using a pH/ORP portable meter (TS-1, Suntex, Taiwan).

### 2.2 MPs and styrene release test

Prior to the simulation experiment, the PS containers and equipment were rinsed with deionized (DI) water. DI water and pH solutions were used for the blank test to confirm the absence of MPs contamination using microscope-equipped FTIR (μ-FTIR). The blank test was performed using the same procedure as that used for experimental sample preparation without MPs. No MPs were detected in the blank test.

The experimental procedure for MPs release from the PS container is shown in Fig. S2. The experiments were performed at different pHs (3, 5, 7, and 9), temperatures (20, 50, 80, and 100 °C), and contact (or exposure) times (2, 4, 6, and 8 h). The pH conditions were simulated based on the pH of the food used in plastic food containers. Temperature was selected based on the European Union (EU) Commission Regulation [41]. The exposure time was set based on a preliminary experiment; the short contact time did not show a clear increasing or decreasing trend in MPs release. No MPs or even very small amounts of MPs were detected. Therefore, a relatively long exposure time was used in this study.

The release tests under different pH, temperature, and exposure time conditions were conducted in the following order. As shown in Fig. S3 (i)∼(iii), experiments were conducted under extreme conditions, the experiment **(ⅳ)** was representing foreseeable general conditions of use as regards contact time. Therefore, the contact time was 30 min at room temperature, and the solution used to represent the food in the test container was pH 5. The pH 5 solution was heated to 100 °C, which is generally used in test containers, and 200 mL of the heated solution was poured into the container.

(i) Effect of pH: A 200 mL of pH (3, 5, 7, and 9) solution in a glass beaker was heated to 100 °C on a hot plate, and each pH solution was poured into PS containers. Each sample was covered with aluminum foil to prevent contamination by other MPs and placed in a preheated oven at a set temperature (100 °C) for a specific time (for 6 h).
(ii) Effect of temperature: The pH 9 solution in which the MPs were detected the most in the preliminary experiment was used to test the effect of the temperature. Similar to the procedure for investigating effect of pH, a 200 mL of pH 9 solution in a glass beaker was heated to 20, 50, 80, and 100 °C using a hot plate, and each solution was poured into the PS containers. Each sample was covered with aluminum foil and placed in a preheated oven at 20, 50, 80, and 100 °C for a specific time (6 h).
(iii) Effect of exposure time: The time experiment was conducted for 2, 4, 6, and 8 h at pH 9 and 100 °C. A 200 mL of pH 9 solution in a glass beaker was heated to 100 °C with a hot plate and was poured into the PS containers. Each sample was covered with aluminum foil and placed in a preheated oven at 100 °C for various durations.

The experiments were repeated at least three times, and the mean values with the corresponding standard deviations are reported.

### 2.3 Identification of microplastics via μ-FTIR

#### 2.3.1 μ-FTIR method validation for microplastics identification

The reliability and accuracy of the μ-FTIR analysis method used in this study were verified according to a previous study [42]. Before the μ-FTIR method was validated, the particle size distributions of PS and PE, the standard materials tha release MPs, were measured. The mean size of PS and PE particles was 258.7 μm and 39.48 μm, respectively (Fig. S4).

**Fig. 1:**
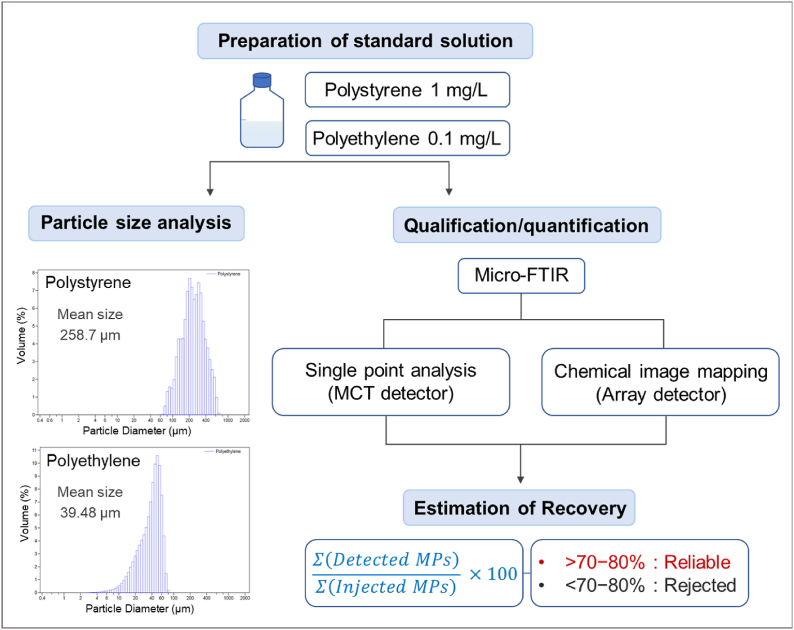
Method validation of MPs qualitative/quantitative analysis.

Based on the qualitative and quantitative analyses of the PS and PE standards described in Section 2.3.2, the identification of MPs in DI water was validated. PS (1 mg L^−1^) with an average particle size of 258.7 μm and 0.1 mg L^−1^ for PE with an average particle size of 39.48 μm were prepared as MPs standards solutions. The standard solution was filtered to deposit PS and PE on an Anodisc filter. Method validation was performed in two steps. First, PS and PE on the Anodisc filter were identified in a single point analysis using an MCT detector and manually counted. Next, chemical image mapping of the manually identified MPs standards was acquired automatically, and the MPs released from PS and PE that exhibited representative IR bands of PS and PE in the reference library were counted. The recovery-determining accuracy and reliability were calculated using the ratio of the manually identified MP numbers to that obtained from chemical image mapping. In the present study, it was assumed that a recovery above 70–80% would be reliable and can be applied for the identification of MP. The PS and PE recoveries were 91.4% and 81.9%, respectively, achieving recovery rates of over 80%. Therefore, it was confirmed that the μ-FTIR chemical imaging mapping used in this study was reliable. The detailed procedure of validation of MPs is shown in Fig. 1.

#### 2.3.2 μ-FTIR analysis for MPs release

After investigating the release of MPs, the entire volume of 200 mL samples was filtered using a vacuum pump through an Anodisc filter (Al_2_O_3_, 0.2 μm, diameter 25 mm, Whatman) to collect the MP particles. To collect any remaining MPs in the glass funnels, they were washed with water. The filtered Anodisc was placed in foil-covered Petri dishes with lids and stored in a desiccator to remove water prior to μ-FTIR analysis.

MPs were identified using a μ-FTIR spectrometer (Nicolet iN10MX, Thermo Fisher Scientific, US). A dried Anodisc filter was placed on the μ-FTIR sample stage, and chemical image mapping analysis was conducted. The number, shape, and maximum diameter of MPs were recorded using a μ-FTIR spectrometer. Four different sizes (10–100, 100–200, 200–300, and >300 μm) were used for comparison. The size and shape of the MPs were measured with an image mapping tool using an FTIR microscope, and images that were visible to the naked eye were recorded.

Before the experiment, method validation was conducted using purchased standard MP materials, PS (powder, BAM-P202, Germany, particle size 206 μm), and PE (powder, Sigma Aldrich, particle size 40–48 μm). The particle size distributions of the standard materials, PS and PE, were measured with a particle size analyzer (PSA, LS 13,320, Beckman Coulter) using the static light scattering method. The particle size distribution was in the range 0.4–2000 μm.

MPs were identified according to a previous study [42] using single-point analysis and chemical image mapping. For single-point analysis, the spectral range was set to 4000–1300 cm^−1^ with a resolution of 4 cm^−1^ and eight scanning times in liquid-nitrogen-cooled transmission mode using a MCT detector.

Chemical image mapping was conducted in the spectral range 4000–1300 cm^−1^ with 16 scans in liquid nitrogen-cooled imaging mode using an array detector that can automatically scan a quarter of the Anodisc filter. Chemical image mapping was performed four times on one Anodisc filter and the data from the four quarters were summed to record the results. The spectra were then compared to the reference spectra of the PS and PE databases of the Thermo Scientific Infrared spectral library. All analyses were performed in triplicate, and MPs were counted after the spectra were acquired from the entire area of the Anodisc with the filtered sample.

### 2.4 Surface morphology of inside PS container via field-emission scanning electron microscope (FE-SEM)

The inner surface of the PS container before and after the MPs release test was observed using field-emission scanning electron microscopy (FE-SEM; Zeiss SUPRA 40VP). The samples were then coated with platinum (Pt). The SEM operating conditions were 100× magnification and accelerating voltage of 10 kV.

### 2.5 Quantification of styrene

#### 2.5.1 Sample preparation for gas chromatography-mass spectrometry (GC-MS) and analysis

After the experiment, the solution in the PS plastic container was adsorbed onto the adsorption tube using the purge-and-trap method. For extraction with a purge-and-trap concentrator, the pH solution was poured into a 30 mL impinger and the solution was aerated in a water base preheated to 50 °C to adsorb styrene, a volatile organic compound, to a Tenax (Markes international) adsorption tube. The mass flow controller (GFC171S, Aalborg) was set to 100 mL min^−1^, the vacuum pump was set to 15 min, and ultra-high-purity nitrogen was used for purging. Fig. S2(c) shows the purge-and-trap and GC-MS analyses of styrene released from the plastic containers.

#### 2.5.2 GC-MS conditions

To quantitatively analyse the styrene released from the plastic containers, an auto-thermal desorber (ATD 650, PerkinElmer, USA) with GC-MS (QP2010 Plus, Shimadzu) was used. A two-stage injection mode was used, and the analysis consisted of three major steps: purging, desorption, and cold trap heating. The purge and trap process can efficiently extract volatile organic compounds, styrene monomer, in this study. The styrene monomers were concentrated onto an absorbent trap and followed by thermal desorption into a gas. First, an adsorption tube was attached to an auto thermal desorber, and heat at 350 °C was applied to perform primary desorption. Then, after concentrating the gas at −40 °C in the secondary tube, it was thermally desorbed in the secondary tube and injected into GC-MS. The automatic thermal desorption machine was linked with the quadrupole type GC-QP-EI-MS, the carrier gas was 99.9999% ultra-high purity helium (He), and the mass range was set to 20–350 *m*/*z*. A GC column (Agilent J&W DB-1, 123–1063) with a 60 m inner diameter of 0.32 mm and polydimethylsiloxane resin coating of 1.0 μm was used. The detector uses the SIM mode to detect only molecules with set mass values (78, 103, and 72 *m*/*z*) to increase the sensitivity of the qualitative analysis of styrene. The retention time was set to 21.5 min–23 min (Fig. S5). Quantitative styrene data were processed using a GC-MS post-run analysis program.

For GC-MS quantitative analysis, a styrene gaseous standard (1 ppm, RESTEK, USA) was used to prepare a calibration curve. The calibration curve (Fig. S6) was prepared by injecting 5, 10, 25, 50, and 100 mL of 1 ppm standard gas into 1 ppm gaseous standard styrene. Fig. S7 shows an overlay of SIM chromatograms of the measured styrene under the designed experimental conditions for *m*/*z* 104 at linearity levels. From the mass spectrum, the base peak at *m*/*z* 104 was used for quantitation, whereas 103, 78, and 72 *m*/*z* were used as reference ions.

### 2.6 Measurement of correlation between released compounds

The correlation of the detected PS-MPs with ethylene glycol monooleate (EGM) or styrene monomers was examined by calculating the correlation coefficients of the substances detected in PS containers under the MPs release condition (2.2). This was performed using the CORREL function in Microsoft Excel (Microsoft EXCEL 2019) [43], which is as follows:

(1) $$Correl\left(X,Y\right)=\frac{\sum \left(x-\bar{x}\right)\left(y-\bar{y}\right)}{\sqrt{\sum {\left(x-\bar{x}\right)}^{2}\sum {\left(y-\bar{y}\right)}^{2}}}$$

A positive (or negative) correlation coefficient calculated using the above equation indicates a positive (or negative) relationship between released amounts of MPs and EGM/styrene under different pH, temperature and exposure time. A linear relationship based on the value of the correlation coefficient was determined according to the criteria listed in Table S1 [44]. A positive correlation occurs when one variable increases with the increase in other variable, and a negative correlation occurs when one variable increases when the other variable decreases.

## 3 Results and discussion

### 3.1 MPs release from PS plastic container under different pH, temperature, and exposure period

**Fig. 2:**
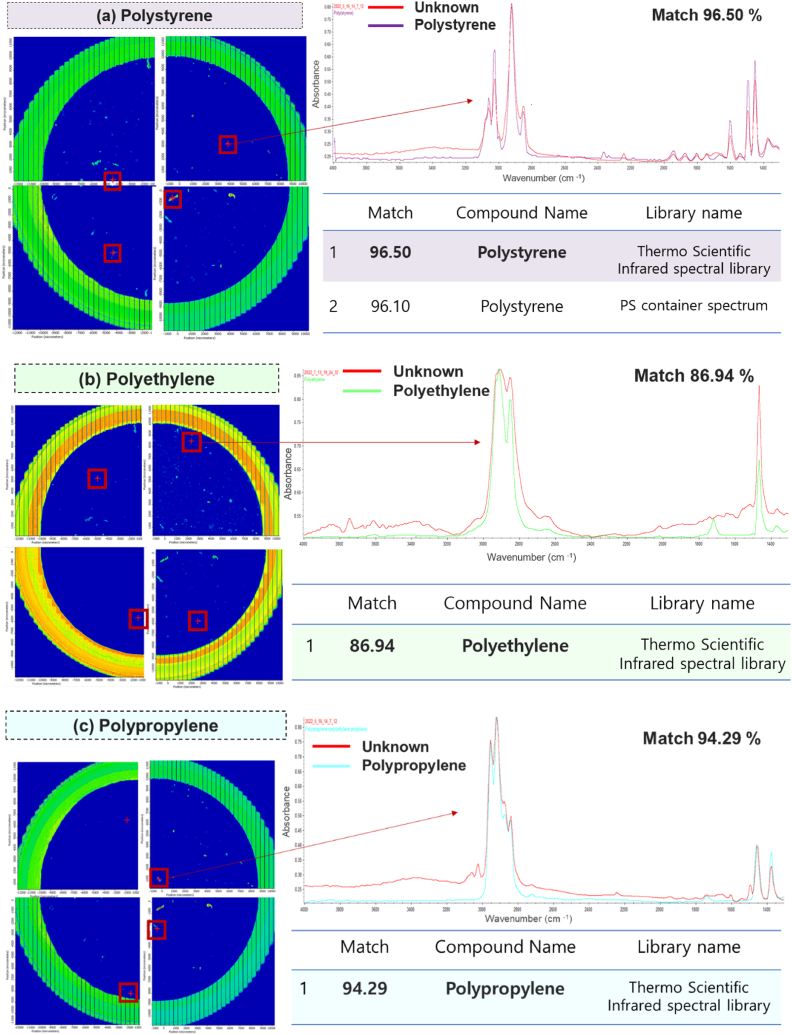
Identification of MPs using chemical image mapping of μ-FTIR analysis: (a) PS, (b) PE, and (c) PP.

MPs can be released under different environmental conditions when disposable PS containers are used. The effects of the pH of the liquid in the container on the release of MPs were systematically examined using a simulated experiment under different pH (acidic, neutral, and basic conditions) and exposure time conditions (2, 4, 6, and 8 h). PS, polyethylene (PE) and polypropylene (PP) MPs were detected in the simulants under each condition, as shown in Fig. 2. The detected PS exhibited strong IR bands at 3062–3023 cm^−1^ (aromatic C–H stretches), 2923–2854 cm^−1^(CH_2_ asymmetric and symmetric stretches), 1596–1489 cm^−1^ (aromatic ring modes), 1450 cm^−1^ (CH_2_ deformation), and 1365 cm^−1^ (CH_3_ deformation) [45]. The detected PE exhibited strong and sharp IR bands at 2918–2854 cm^−1^ (CH_2_ stretching band) and 1467 cm^−1^ (C–H stretching) [46]. The PP IR spectra was observed at 2949 cm^−1^ (CH_3_ stretching band), 2917 cm^−1^ (CH_2_ stretching band), 2865 cm^−1^ (CH_3_ stretching band), 2835 cm^−1^ (C–H stretches), 1456 cm^−1^ (CH_3_ symmetrical bending), and 1371 cm^−1^ (CH_3_ symmetrical bending) [[47], [48], [49]].

**Fig. 3:**
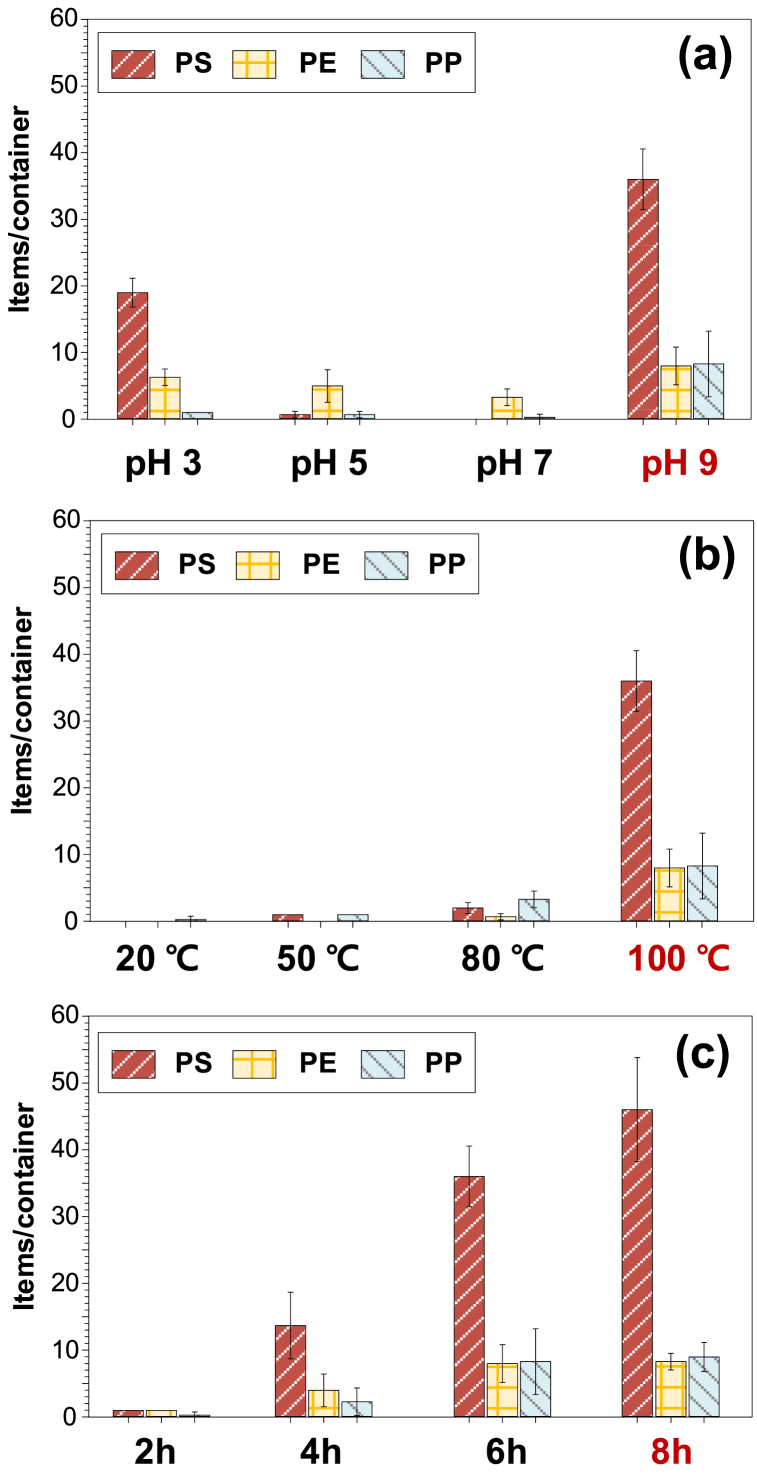
Number of MPs released from PS container (a) at different pH values (3, 5, 7, and 9) (6 h and 100 °C), (b) at different temperatures (20, 50, 80, and 100 °C) (pH 9 and 6 h) (pH 9 and 8 h), and (c) at different times (2, 4, 6, and 8 h) (pH 9 and 100 °C).

Overall, high amounts of PS-MPs were released at pH 3 and 9, and the release of MPs was directly proportional to temperature and exposure time. The pH of the experiment conducted for detecting temperature and time was set at 9 because the highest amounts of PS microplastics were detected at pH 9. In particular, the highest number of PS-MPs (36 particles/container) was released under alkaline conditions (pH 9) at 100 °C for 6 h (Fig. 3(a)), which was two-fold higher than that under acidic conditions (pH 3). In contrast, PS-MPs were barely detectable at pH 5 or 7. PS-MPs were not detected at 20 °C, and only small number of PS-MPs were detected at 50 and 80 °C. When the temperature increased from 80 °C to 100 °C, the number of detected PS-MPs increased substantially (Fig. 3(b)). MPs were hardly detected after 2 h, and the amount of MPs gradually increased after 4 h of exposure at 100 °C (Fig. 3(c)). When the time increased to 6 h, two-folds the number of PS-MPs than that at 4 h was detected. When the exposure time increased from 6 h to 8 h, the number of PS-MPs increased by 28%. After 10 h, the experiment could no longer be conducted owing to the severe deformation of the plastic container. This indicated that the basic condition, high temperatures (100 °C) and long duration (>4 h) was the main factor affecting the release of PS-MPs in the PS container.

Under acidic and basic conditions, abundant H^+^ and OH^−^ ions may contribute to the oxidation process, which results in the fragmentation of PS particles in the MPs size. PS, which is a polymer with C–C backbone, is susceptible to thermal oxidation. The expanded PS when used as a material for disposable food containers was exposed at softening temperature of 90 °C; at this temperature, a needle penetrates to the depth of 1 mm of the specimen. In addition, the synergistic effect of high temperature (100 °C) and abundant OH^−^ ions may accelerate the highest amount of fragmentation of PS container into MPs size.

Interestingly, although PS was the main component of the containers, noticeable amounts of PE and PP were detected. The source of the PE-MPs was carefully examined by characterization of the surface of the PS container using ATR-FTIR. As shown in Fig. S1(b), no any IR band of PE was detected. IR band of PE is overlayed with that of PS at 2918–2854 cm^−1^ (CH_2_ stretching band) and 1467 cm^−1^ (C–H stretching). The interference of PE spectrum by PS is induced when the proportion of PE is less than 5%. It indicates that a mere amount of PE presents as a plasticizer that functions for contacting with foods. PE and PP are commonly used in food packaging to prevent direct contact with food [50,51]. Previous studies have also detected the presence of different MPs in plastic containers, suggesting that the differences in MP content between different types of plastic containers may be related to different material properties owing to different manufacturing processes [17,52]. Similar to PS, PE release was proportional to the temperature and exposure time, but the amount released was three to five times smaller than that of PS. However, no distinct effect of pH on PE release was observed. This indicated that high temperature and long duration were the main factors affecting PE release. As the temperature increased, the amount of PS-MPs released increased, which was similar to the results obtained by Guan et al. [53]. This was probably because chain scission occurs more easily and the polymer structure decomposes as the temperature increased [54].

Furthermore, the measurement of the size of MPs is particularly important for human health risk assessment. Although MPs larger than 150 μm are unlikely to be absorbed by the human body, smaller MPs (<150 μm) may lead to systemic exposure [55]. The sizes of the PS-MPs, PE, and PE/PP-MPs were predominantly 10–100 μm (Fig. S8 (a), (b) and (c)), indicating that PS-MPs released from PS containers may be transported to the human body via ingestion.

The shape of the PS-MPs is shown in Fig. S9. Fragments were mostly observed as MPs shape. Primary plastics are produced and added to the daily products, thus have a lamellar or spherical shape [56]. Meanwhile, secondary MPs that were released after fragmentation process by human activities have irregular shape-fragment type. Thus, it assumes that the PS-MPs were fragmented into small size under corresponding conditions and released into the liquid simulants.

The surface of the PS container was observed using FE-SEM to examine the release of the PS-MPs and EGM. Fig. S10(a) shows the initial state of the PS container inside (control) and Fig. S6b–e shows the images of the surface inside the PS container after the pH experiment. Compared to the initial state, the swelling spots were broken and opened at pH values of 3 and 9, corresponding to the increase in MPs release under these conditions and the high amounts of released PS-MPs at these temperatures. Similarly, broken and opened swelling spots were observed as the exposure to pH at 100 °C continued for 4 and 8 h (Fig. S8 (g), (h)). However, there was no noticeable change in the inner surface with an increase in temperature up to 80 °C (Fig. S8 (i), (j), (k)). It was assumed that MPs were released after the surface of the container burst when the exposure duration increased to 8 h and temperature to 100 °C under pH 9.

### 3.2 Simultaneously exposed contaminants with PS-MPs

#### 3.2.1 Ethylene glycol monooleate (EGM)

**Fig. 4:**
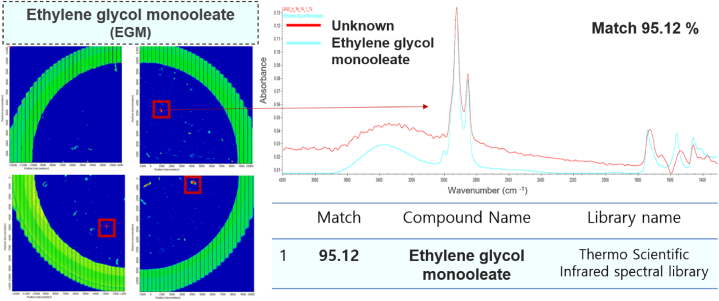
Identification of EGM using chemical image mapping of μ-FTIR analysis.

Plastics consist of plasticisers, monomers, and derivatives added during the manufacturing process. We assumed that these substances can be released simultaneously with MPs and exposed to humans who ingest food and/or beverages in PS containers. The identified EGM showed strong peaks at 2927–2854 cm^−1^ (C–H stretching), 1740 cm^−1^ (C=O stretching) and 1465 cm^−1^ (C–H bending) [49]; this observation was consistent with more than 90% of the EGM in the reference library (library name; Thermo Scientific Infrared spectral library-coating technology, OMNIC software, Thermo Fisher Scientific) (Fig. 4). EGM is mainly used as a preservative, resin plasticizer, emollient, and thickener in the food and pharmaceutical industries because of its low price, low toxicity, volatility, and biodegradability [57,58].

**Fig. 5:**
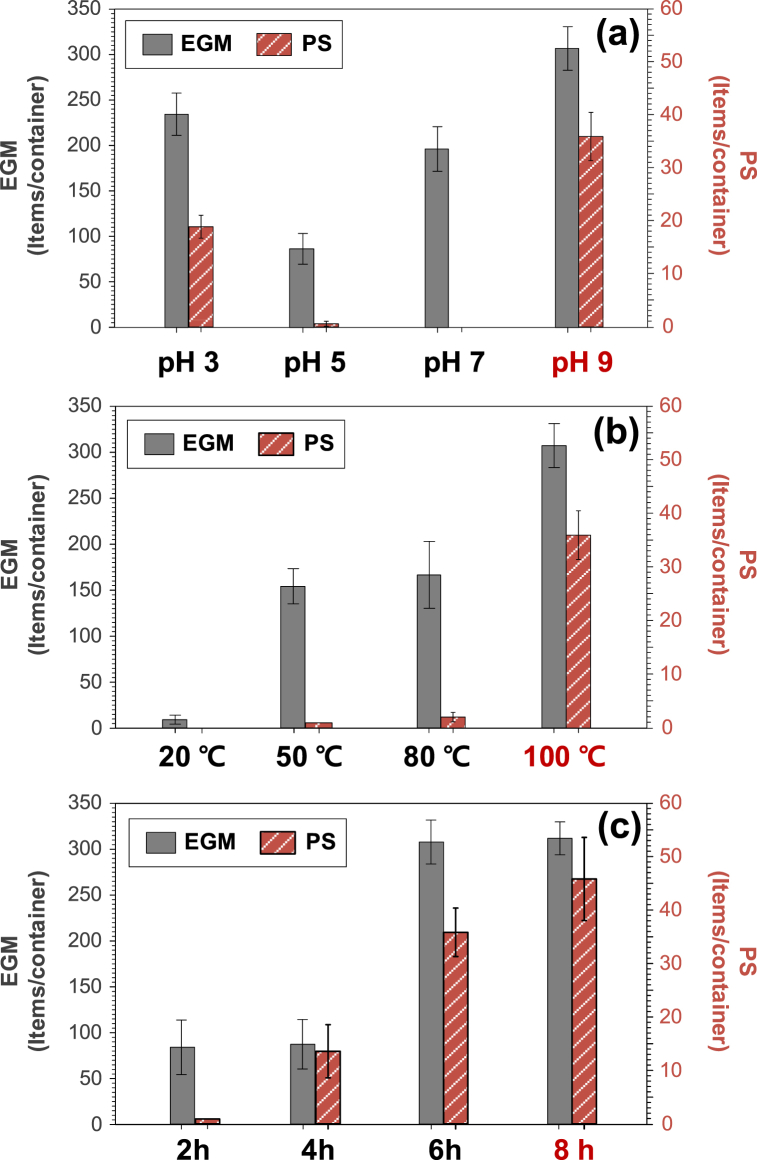
(a) Number of EGMs released from PS container at different pH values (3, 5, 7, and 9) (6 h and 100 °C), (b) Number of EGMs released at different temperatures (20, 50, 80, and 100 °C) (pH 9 and 6 h), and (c) Number of EGMs released at different times (2, 4, 6, and 8 h) (pH 9 and 100 °C).

Overall, 10 times higher numbers of ethylene glycol monooleate (EGM) were identified as the major SEPs under all conditions of the MPs release test (Fig. 5). The highest numbers of EGM were released at pH 9 and 100 °C after 8 h of exposure time. The number released at pH 9 was proportional to temperature and exposure time (Fig. 5(b), (c)). In contrast to PS-MPs, considerable numbers of EGM were simultaneously released at low pH (3 and 5) and neutral pH (7) (Fig. 5(a)). High numbers of EGM were released within 2 h (Fig. 5(c)). This indicates that EGM is more easily released into the liquid in the PS containers than PS-MPs. High numbers of EGM were released even at a mildly increasing temperature (50 °C) (Fig. 5(b)), while only a small number of PS-MPs was released under the corresponding conditions. The majority of the EGM was in the range of 10–200 μm, as shown in Fig. S8(d).

We carefully examined the presence of EGM in the PS container. EGMs has glycerol with fatty acid, which having IR bands of O─H at 3400 cm^−1^, CH_2_─CH_2_ at 2923–2854 cm^−1^, and C=O (ester) at 1790 cm^−1^ (Fig. 4).

The glycerol monooleate is a substance of slip agent, which is solubilized in the amorphous melt, and consequently forming lubricant layer. It is added to polymers to control friction of the plastic product. In the release test, the EGM migrated into the water in the PS container. As shown in Fig. S1, PS was identified in the PS container via ATR-FTIR analysis and EGM was not detected. This means that the inner surface of the PS container predominantly consisted of PS. Small portions of EGM are assumed to exist as a mixture of PS. However, the EGM is different from plastic particles, thus were not classified as MPs. While MPs are fragmented plastics from the bulk materials (PS containers), EGM is one of the chemical substances that were migrated from the bulk plastics. Hence, quantification of EGM should be differentiated with MPs. In this study, EGM migration into food simulants provides a good indication of health risk assessment of disposable plastic containers.

#### 3.2.2 Styrene monomer

Plastic monomers are critical components that can be released simultaneously with MPs at the pH, temperature, and time. Styrene is associated with neurotoxicity in humans, and exposure to styrene by inhalation affects the nervous system [59]. Subtle but significant organic psychiatric disorders have been reported in subjects exposed to low levels [60], and another study has revealed the potential for hepatotoxicity [61]. Because styrene has direct negative effects on human health, it is important to study styrene release conditions. Styrene migration from the PS container was examined at various pH values, temperatures, and times using GC-MS.

To quantify the migration of styrene to liquid food simulants, the partitioning of styrene between octanol and water (Log P_ow_) should be examined. 2.70 as log P_oct/water_ means that styrene migrates more easily to octanol, representing a hydrophobic solvent. In this study, food simulants were set up in DI water without any oil substances; thus, the measured styrene amounts were smaller than the actual amounts that originally migrated from the PS container under the simulated conditions.

**Fig. 6:**
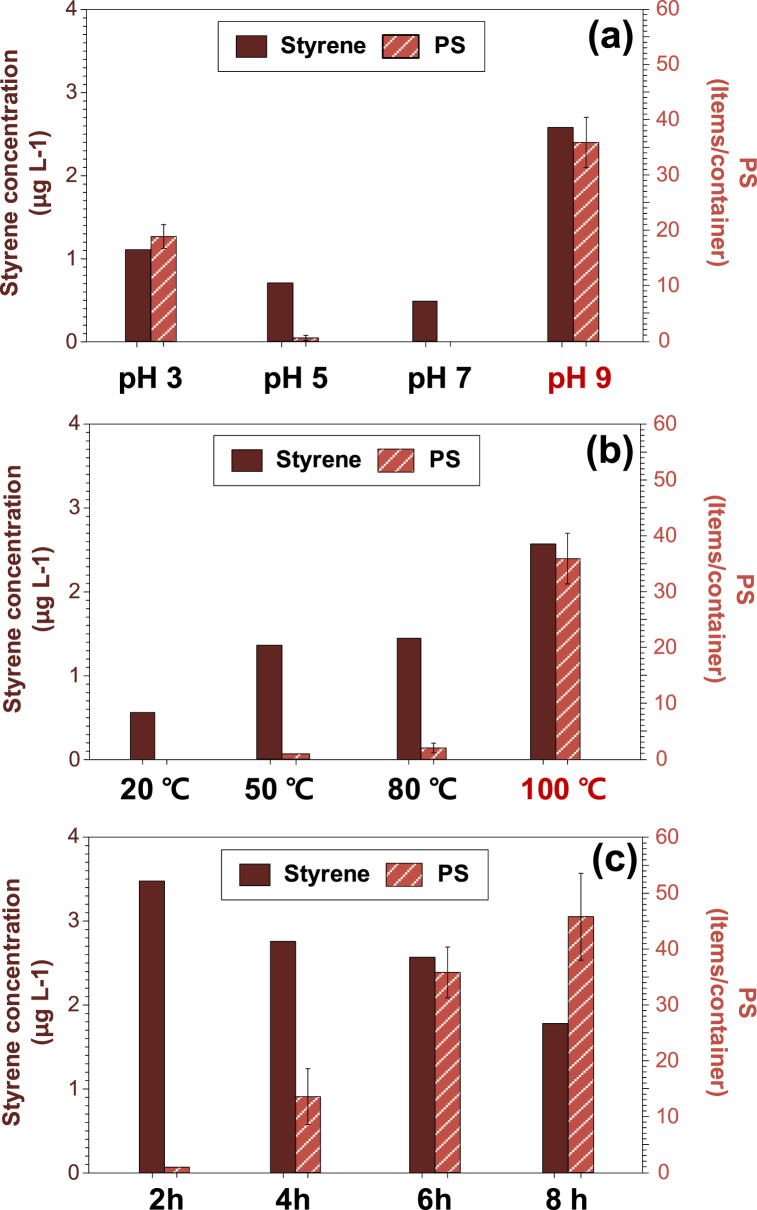
Migration of styrene (a) at different pH values (3, 5, 7, and 9) (6 h and 100 °C), (b) at different temperatures (20, 50, 80, and 100 °C) (pH 9 and 6 h), and (c) at different times (2, 4, 6, and 8 h) (pH 9 and 100 °C).

As shown in Fig. 6(a), styrene migration from the PS container was the highest (2.58 μg/L) at pH 9, whereas 1 and less than 1 g/L of styrene were released at pH 3, 5, and 7, respectively. Under alkali and acidic conditions, abundant OH^−^ and H^+^ ions accelerate styrene migration from polystyrene by attacking C─H bonds and forming H_2_O and/or H_2_. At pH 9, styrene migration increased up to 100 °C (Fig. 6 (b)), but decreased as the exposure time increased (Fig. 6 (c)). This corresponds to a previous study showing that styrene migration was directly proportional to the increase at a specific temperature and exposure time, based on the diffusion coefficient of styrene monomers via Fick’s law [9]. However, in this study, styrene decreased from 2 h to 8 h in the release test. It is presumably due to longer exposed time (4–8 h) in this study than 1 h in the previous one. The exposure time of up to 8 h was set to that for the release of PS-MPs (Fig. 3).

### 3.3 MPs release mechanisms

#### 3.3.1 Correlation between MPs and simultaneously released substances from the PS container

**Fig. 7:**
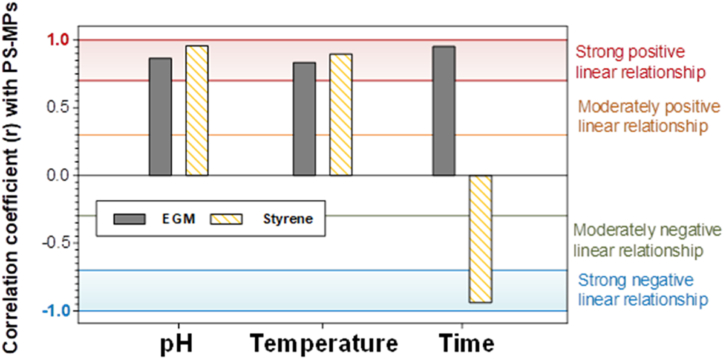
Correlation coefficients (r) between PS-MPs and EGM/styrene concentrations under different pH, temperature and exposed time conditions.

The correlation between MPs release and SEPs was examined to understand the MPs release mechanisms in the PS container. The release under different pH values and temperatures was strongly positively correlated with EGM and styrene monomer, corresponding to the correlation coefficient (>0.7), as shown in Fig. 7 and Table S3. This indicates that the release of MPs and EGM is governed by the same process in the PS container. The plastic fragmentation process is controlled by different pH and temperature conditions because of the physicochemical properties of the plastics. Under acidic and basic conditions (H^+^ and OH^−^ abundance), hydrolysis and oxidation processes occur, resulting in the unchaining of the polymer and fragmentation into smaller sizes. This accelerated as the exposure time at the pH and temperature conditions increased. Meanwhile, styrene release was strongly negatively correlated with PS-MPs release as exposure time increased. This means that styrene migration follows the same process as the PS fragmentation process by pH and temperature change, but is inversely affected as the exposure time increases. This is probably due to the relatively low partitioning of styrene to water (logP_oct_ 2.70) and equilibrium of styrene concentration in the liquid simulants [38]. The maximum concentration of migrated styrene might have been reached within 2 h of the release test. Thus, from 2 h to 8 h, the concentration gradually decreased.

#### 3.3.2 PS release mechanisms

**Fig. 8:**
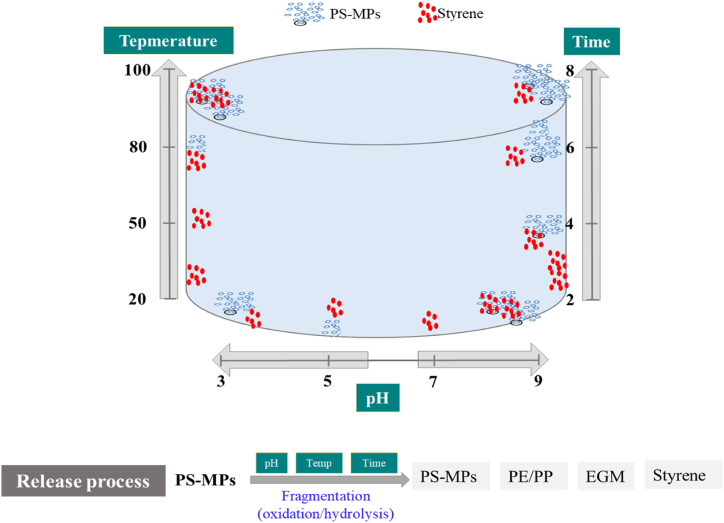
Release mechanism of PS-MPs and simultaneously exposed pollutants (SEPs).

The effects of pH, temperature, and exposure time on the release of MPs and SEPs indicate their release mechanisms. PS fragmentation into smaller particles proceeds by oxidation/hydrolysis under acidic and basic conditions. Interestingly, PE/PP and EGMs, as plastic additives, were also released by the same fragmentation process. Increasing the temperature up to the critical point of PS fragmentation even accelerates the progress, consequently increasing the release of PS-MPs and SEPs as the exposure time increased. However, the styrene monomer migration followed a different release process as the exposure time increased because its migration to the liquid simulant (water) was controlled by the partition coefficient of PS. A schematic of the release mechanism at different pH values, temperatures, and exposure times is shown in Fig. 8.

## 4 Conclusions

MPs release from PS container was the highest at base conditions, because PS fragmentation into MPs size was proceeded by oxidation/hydrolysis and induced by temperature and exposed time. The existence of SEPs at MPs release test implies that humans are being exposed to the unwanted and unknown amounts of pollutants that may potentially threaten human health. Prior to standard regulation on MPs and SEPs release from daily products, their quantification and qualification should be established in a systematical way. Further studies are needed on the MPs and SEPs release under oil contained food simulants together with their release mechanisms at this condition.

## 5 Environmental implication

As the usage of consumable plastic container is increasing extensively, disposal of the used containers generates serious problems in aquatic and soil environments. We assumed that plastic containers could generate considerable amounts of microplastics in aquatic and soil environments. We hypothesised that differences in the amounts of MPs would exist under different usage conditions and set up four different release tests to identify them.

This study revealed that the highest amount of MPs was released at pH 9. Interestingly, simultaneously exposed pollutants-polyethylene, ethylene glycol monomer, and styrene were detected under the same conditions. It would be significantly increasing if it were broadened to global consumption.

## Author contribution statement

Jiae Wang: Conceived and designed the experiments; Performed the experiments; Analyzed and interpreted the data; Wrote the paper.

Jieun Lee: Conceived and designed the experiments; Analyzed and interpreted the data; Contributed reagents, materials, analysis tools or data; Wrote the paper.

Eilhann E. Kwon: Conceived and designed the experiments; Performed the experiments; Contributed reagents, materials, analysis tools or data; Wrote the paper.

Sanghyun Jeong: Conceived and designed the experiments; Analyzed and interpreted the data; Contributed reagents, materials, analysis tools or data; Wrote the paper.

## Data availability statement

No data was used for the research described in the article.

## Nomenclature

EGM: Ethylene glycol monooleate
FE-SEM: Field-emission scanning electron microscope
FTIR: Fourier-transformed infrared spectroscopy
GC-MS: Gas chromatography-mass spectrometry
MPs: Microplastics
PE: Polyethylene
PP: Polypropylene
PS: Polystyrene
SEP: Simultaneously exposed pollutant
