# Supplementary material for: Quantitative analysis of polystyrene microplastic and styrene monomer released from plastic food containers
Source: Heliyon. 2023 Apr 25;9(5):e15787. doi: 10.1016/j.heliyon.2023.e15787 (PMC10256855; doi:10.1016/j.heliyon.2023.e15787)
Supplement: Multimedia component 1 [file mmc1.docx]

# **Supplementary Information**

**Quantitative analysis of polystyrene microplastic and styrene monomer released from plastic food containers**

Jiae Wang^1^, Jieun Lee^2*^, Eilhann E. Kwon^3^, Sanghyun Jeong^1*^

^1^Department of Environmental Engineering, Pusan National University, Busan 46241, South Korea

^2^Institute for Environmental and Energy, Pusan National University, Busan 46241, South Korea

^3^ Department of Earth Resources and Environmental Engineering, Hanyang University, Seoul 04763, the Republic of Korea

^*^Corresponding authors: [99atkins07@pusan.ac.kr](mailto:99atkins07@pusan.ac.kr) (Jieun Lee), sh.jeong@pusan.ac.kr (Sanghyun Jeong)

Table S1. Criteria of correlation relationship.

| **Correlation coefficient (r)** | **Interpretation** |
| --- | --- |
| - 1 < r < - 0.7 | Strong negative linear relationship |
| - 0.7 < r < - 0.3 | Moderately negative linear relationship |
| - 0.1 < r < 0.1 | Negligible |
| 0.1 < r < 0.3 | Weakly positive linear relationship |
| 0.3 < r < 0.7 | Moderately positive linear relationship |
| 0.7 < r <1 | Strong positive linear relationship |

Table S2. Summary of styrene quantitative analysis results.

| **No.** | **Sample** | **Styrene concentration (μg L^-1^)** |
| --- | --- | --- |
| 1 | pH 3 | 1.11 |
| 2 | pH 5 | 0.71 |
| 3 | pH 7 | 0.49 |
| 4 | pH 9 | 2.58 |
| 5 | 20℃ | 0.56 |
| 6 | 50℃ | 1.37 |
| 7 | 80℃ | 1.45 |
| 8 | 100℃ | 2.58 |
| 9 | 2 h | 3.49 |
| 10 | 4 h | 2.77 |
| 11 | 6 h | 2.58 |
| 12 | 8 h | 1.79 |

Table S3. Correlation coefficients (r) between PS-MPs abundance and EGM abundance/styrene concentrations.

| **Conditions** | **Correlation coefficient (r) with PS-MPs** | |
| --- | --- | --- |
|  | **EGM** | **Styrene** |
| **pH** | 0.8643 | 0.9579 |
| **Temperature** | 0.8334 | 0.8950 |
| **Time** | 0.9519 | - 0.9385 |


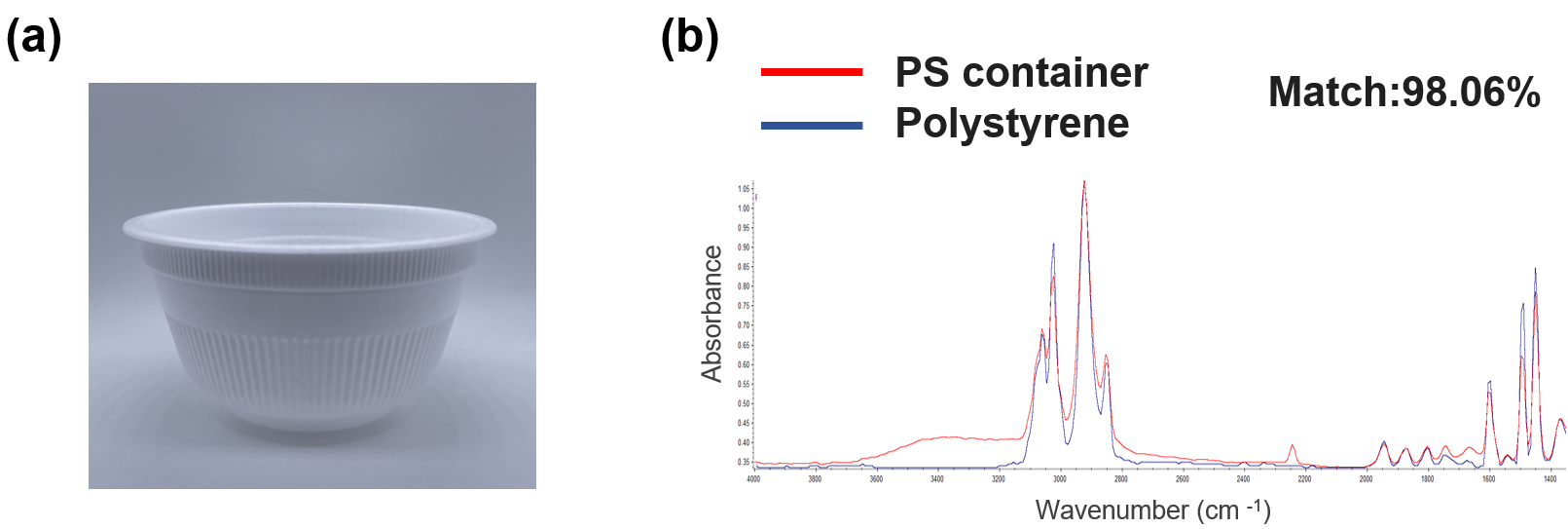


Fig. S1. (a) a picture of the PS food container used and (b) spectra of PS food container (red) and PS container matched 98.06% with the PS database (blue).

| 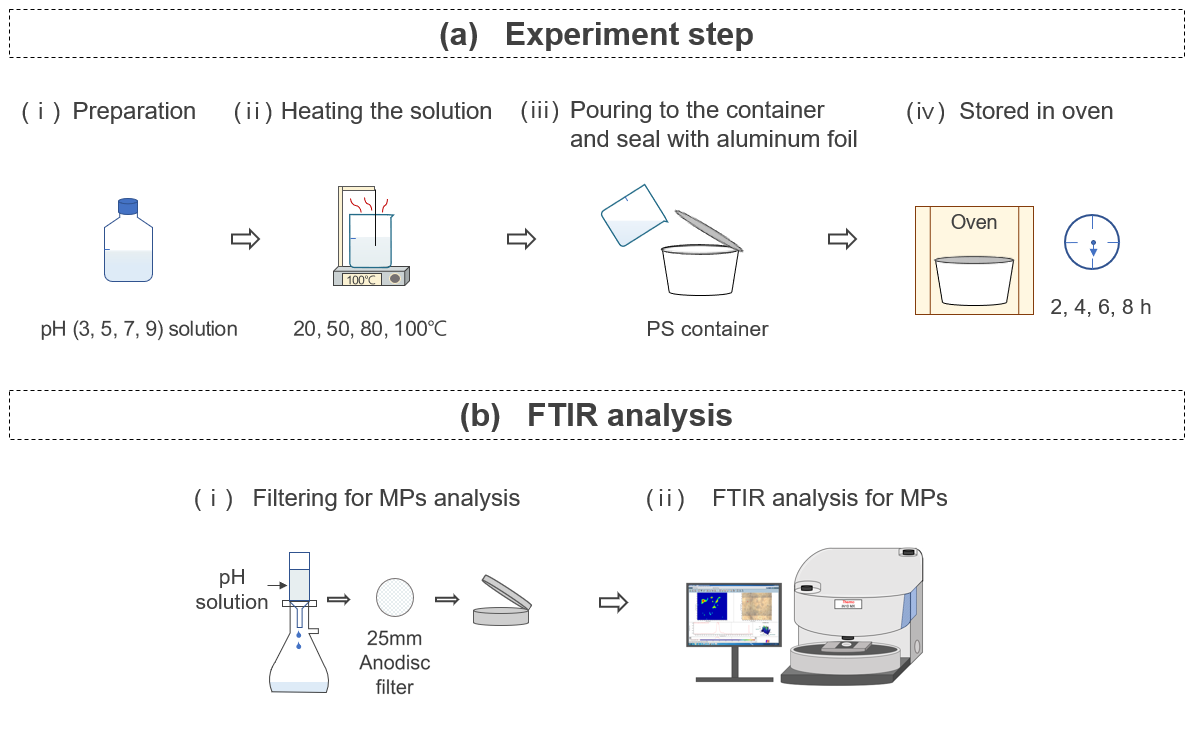 |
| --- |
| 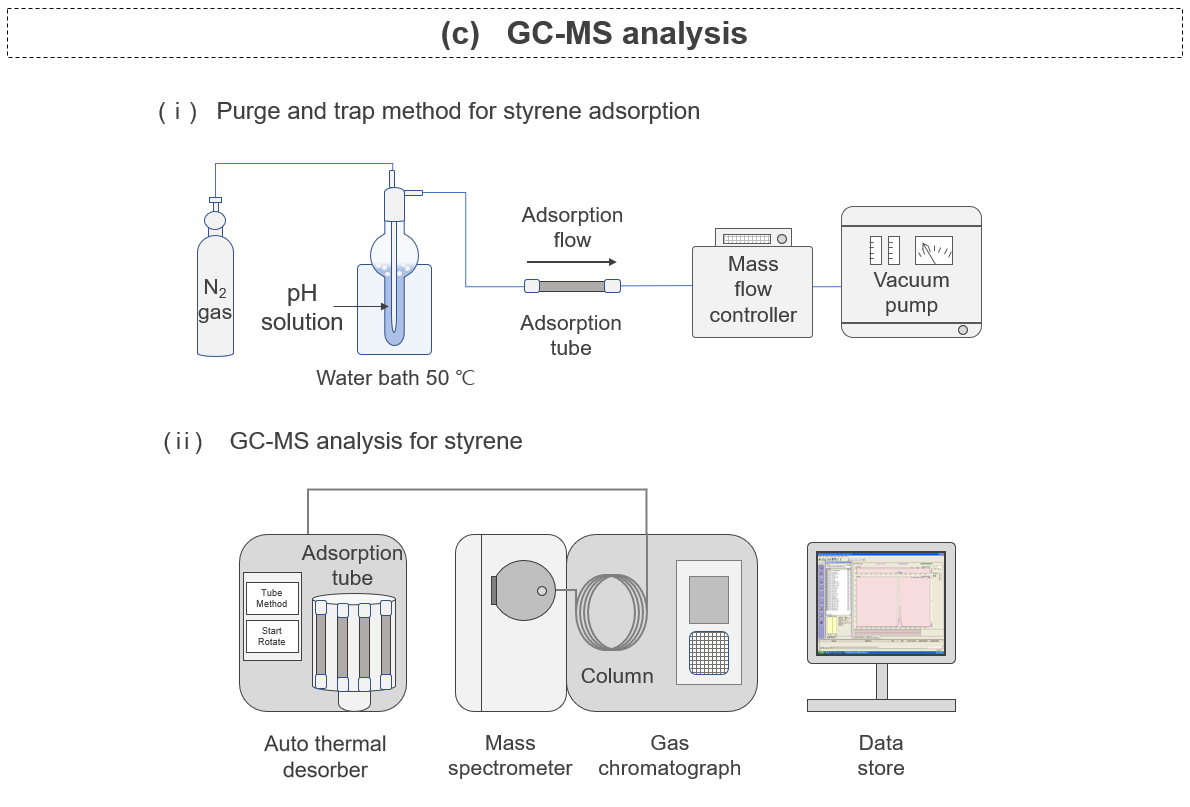 |

**Fig. S2**. Overall experimental procedure of release test and analysis of MPs and styrene from plastic container: (a) Release experimental set-up, (b) FT-IR analysis and (c) GC-MS analysis.

**
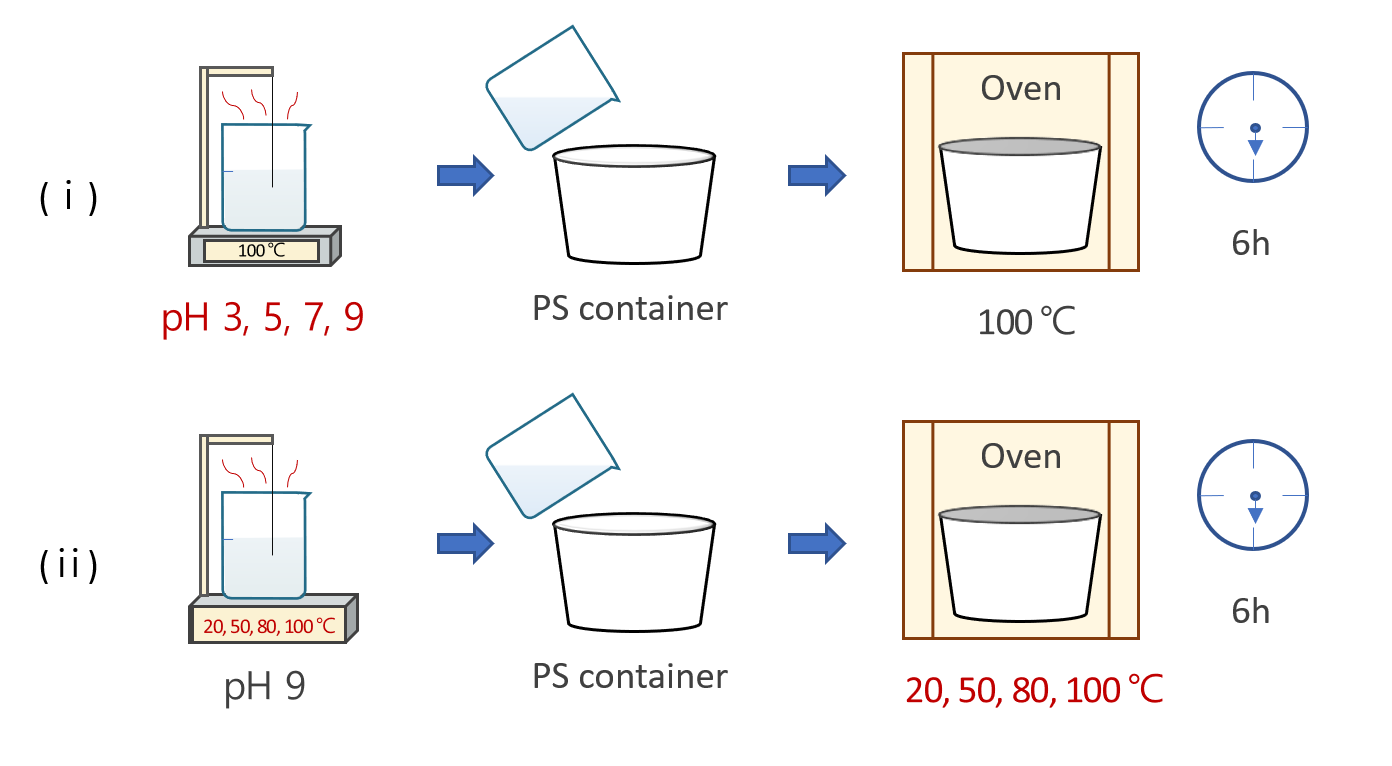

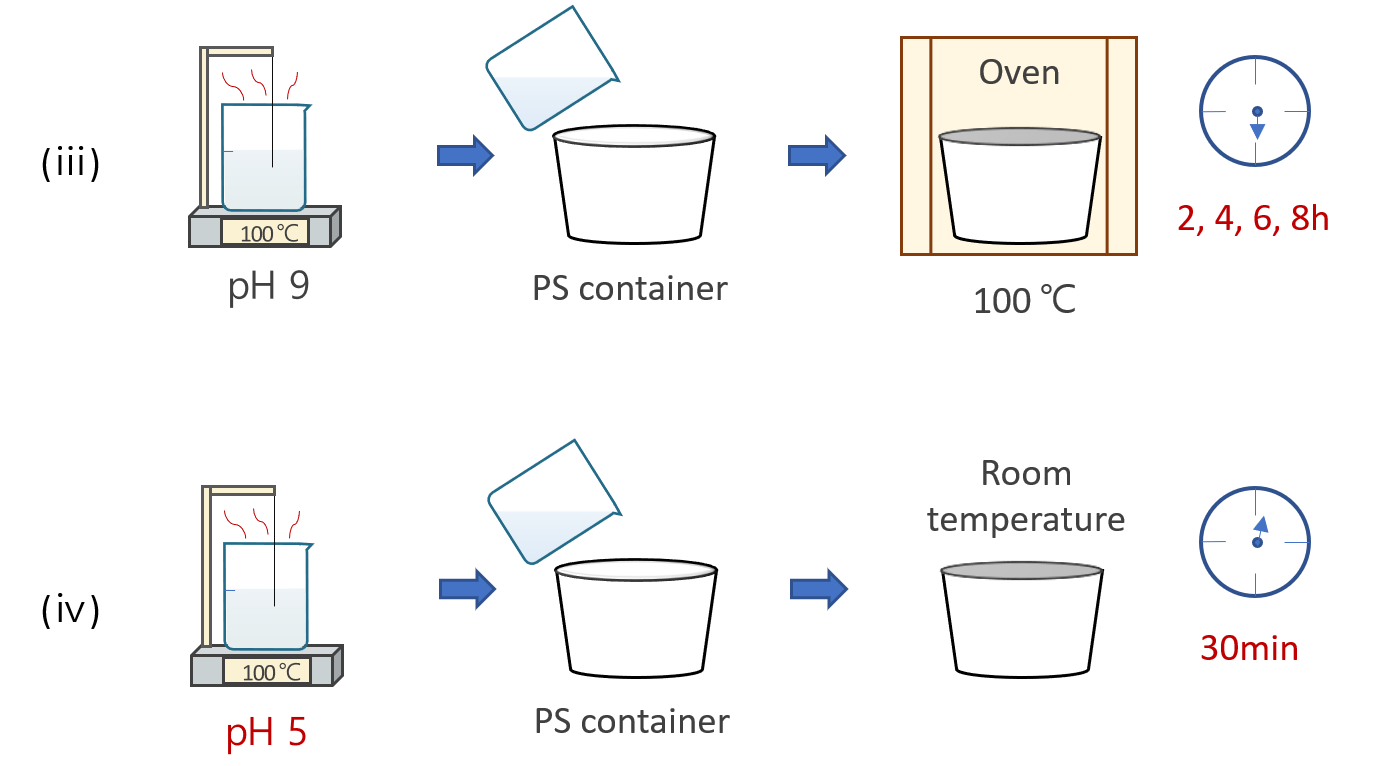
**

Fig. S3. Detailed experimental procedure of release test and analysis of MPs and styrene from plastic container: (i) different pH values (3, 5, 7, and 9) (6 h and 100℃), (ⅱ) different temperatures (20, 50, 80, and 100℃) (pH 9 and 6 h), (ⅲ) different times (2, 4, 6, and 8 h) (pH 9 and 100℃), and (ⅳ) general conditions (pH 5, room temperature, 30 min).

| 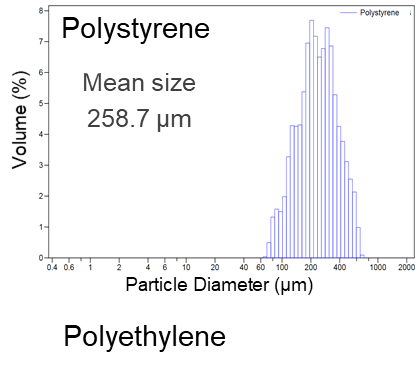 |
| --- |
| 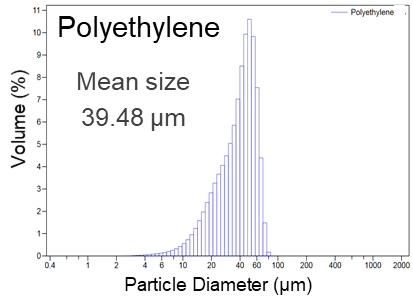 |

Fig. S4. Particle size distribution of PS (top) and PE (down).

Fig. S5. SIM chromatograms of styrene released at general conditions (pH 5, room temperature, 30 min).

Fig. S6. Calibration curve for Styrene.

Fig. S7. SIM chromatograms: (a) Styrene released from PS container at different pH values (3, 5, 7, and 9) (6 h and 100℃), (b) Styrene released at different temperatures (20, 50, 80, and 100℃) (pH 9 and 6 h), and (c) Styrene released at different times (2, 4, 6, and 8 h) (pH 9 and 100℃).


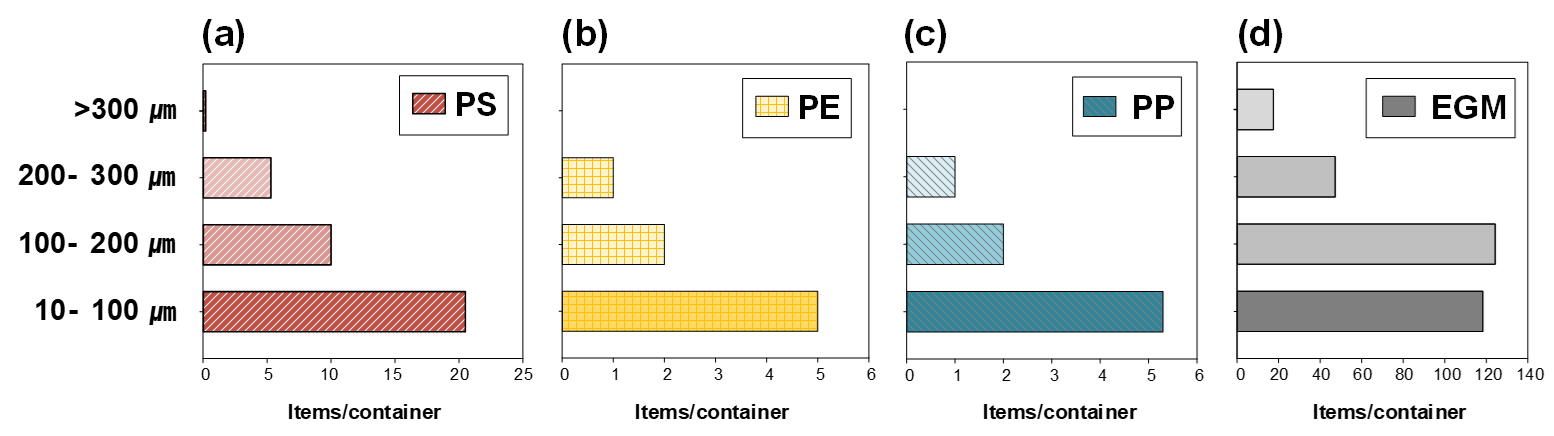


Fig. S8 Size distribution of (a) PS, (b) PE, and (c) PP and (d) EGM (pH 9, 6h, and 100℃).


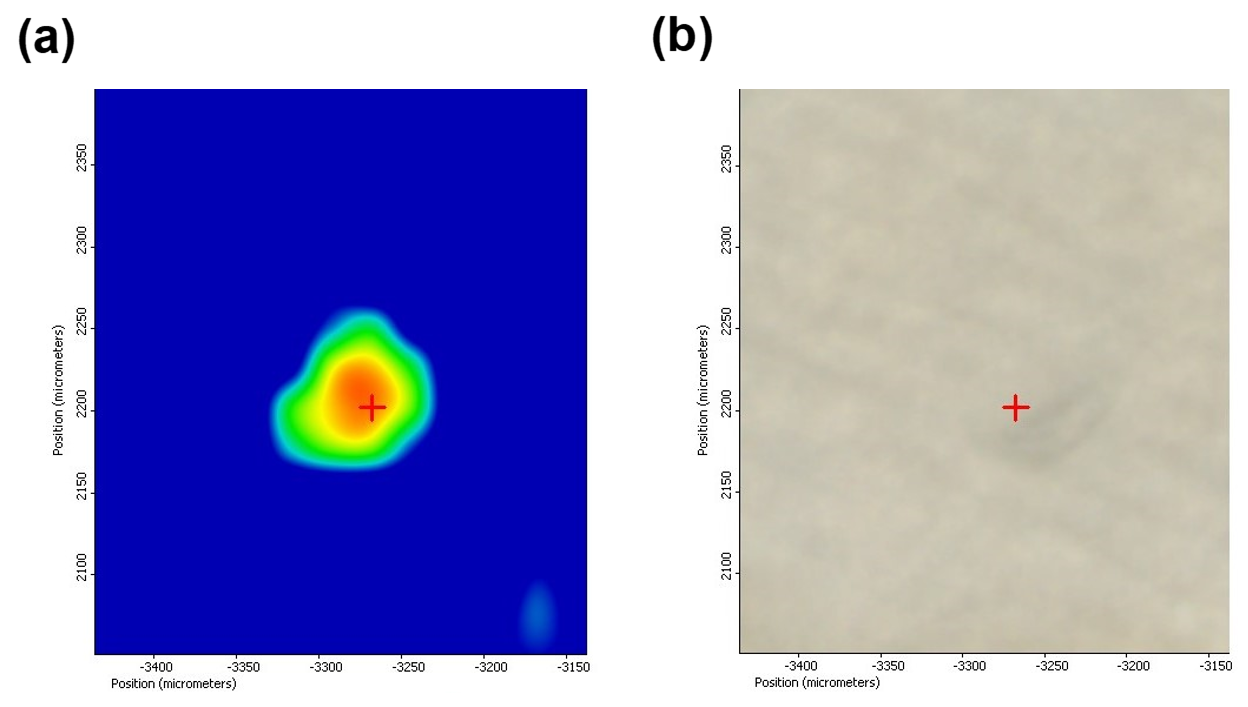


Fig. S9 Observation of MPs shape using μ-FTIR analysis (a) chemical image mapping and (b) microscopic images of μ-FTIR (red color indicates that the IR spectrum in the corresponding spot has more than 80 score of the PE reference IR spectrum).


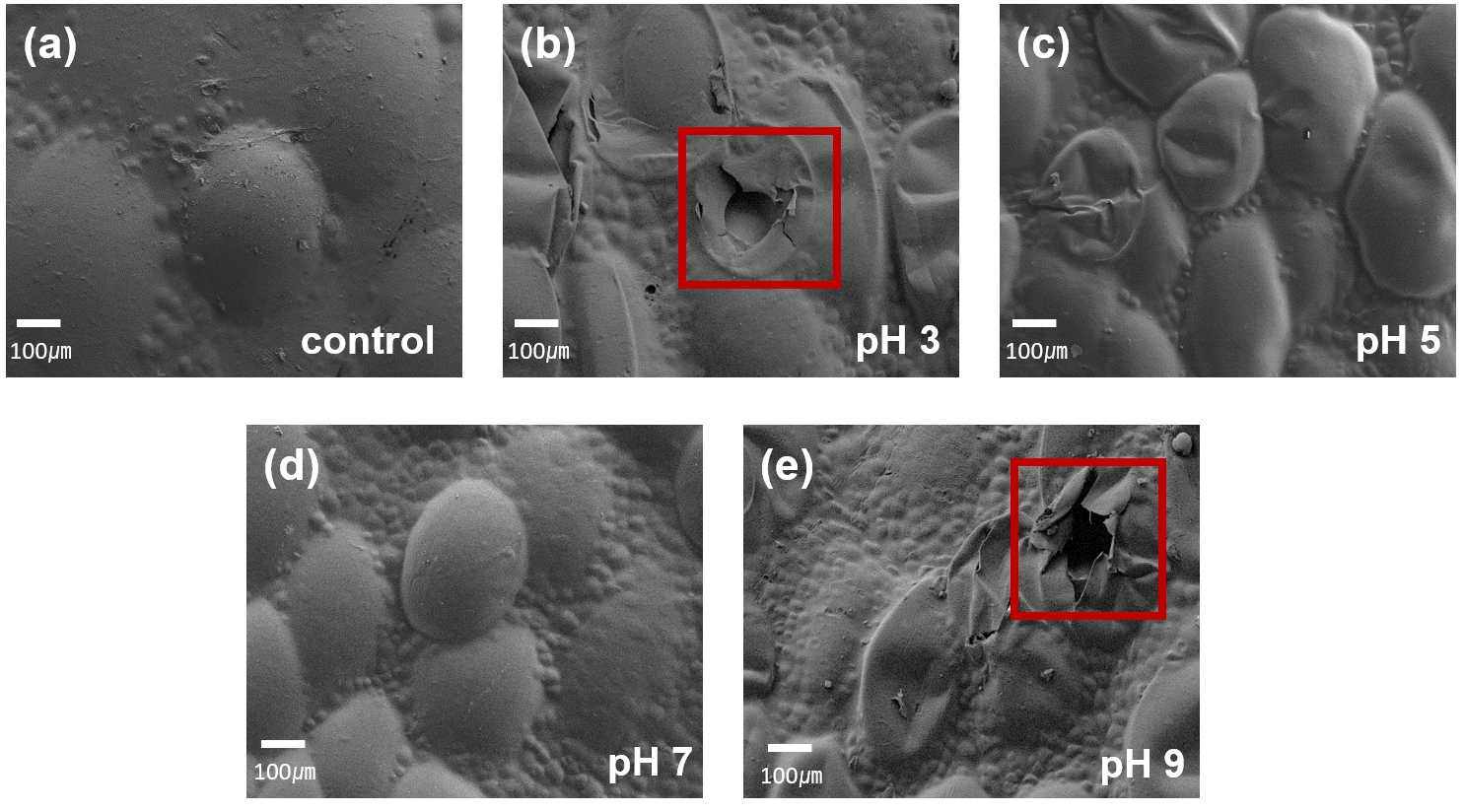


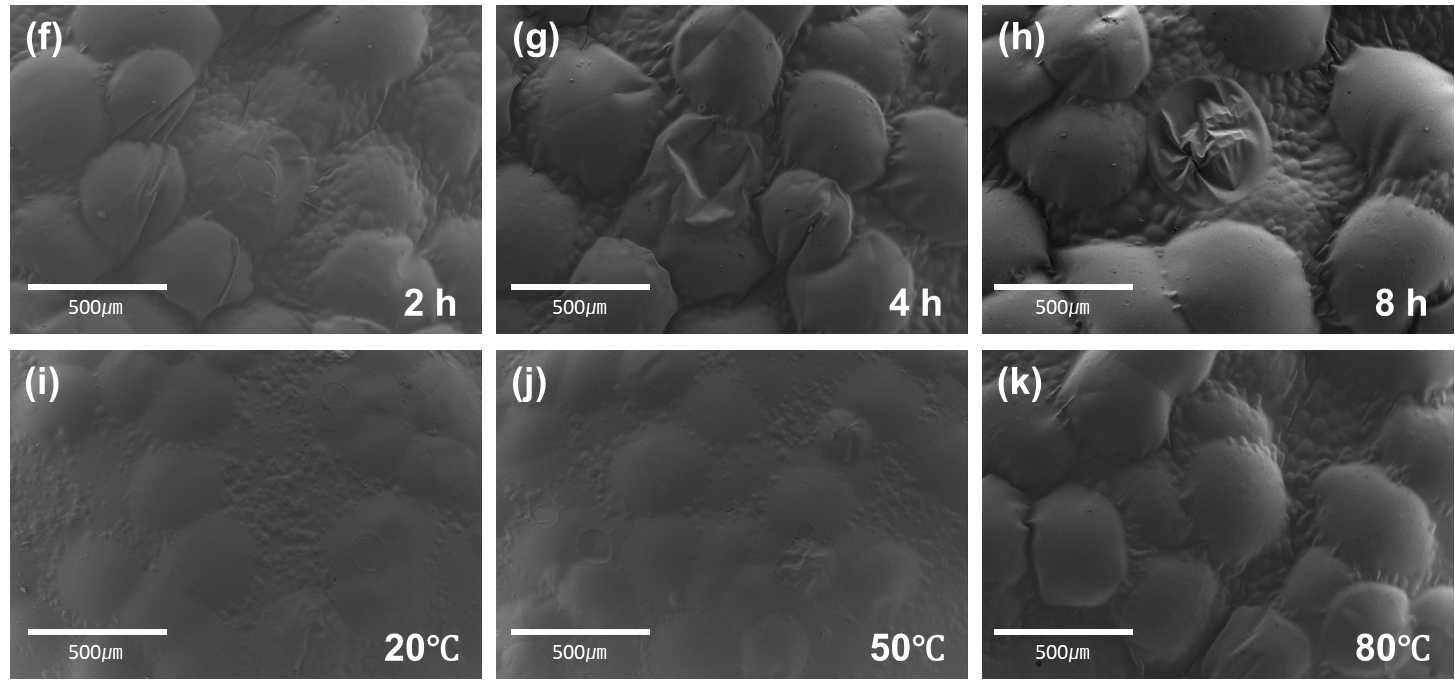


Fig. S10 SEM images of the inner surface of the PS plastic containers at (a) control and (b-e) pH 3- 9 and under pH 9 conditions: (f-h) exposed time from 2 to 8 h and (i-k) temperature from 20 to 80 ºC.
